# First-passage times and normal tissue complication probabilities in the limit of large populations

**DOI:** 10.1038/s41598-020-64618-9

**Published:** 2020-05-29

**Authors:** Peter G. Hufton, Elizabeth Buckingham-Jeffery, Tobias Galla

**Affiliations:** 1 Theoretical Physics, School of Physics and Astronomy, The University of Manchester, Manchester, M13 9PL United Kingdom; 2 School of Mathematics, The University of Manchester, Manchester, M13 9PL United Kingdom; 3 Instituto de Física Interdisciplinar y Sistemas Complejos IFISC (CSIC-UIB), 07122 Palma de Mallorca, Spain

**Keywords:** Biological physics, Cancer models

## Abstract

The time of a stochastic process first passing through a boundary is important to many diverse applications. However, we can rarely compute the analytical distribution of these first-passage times. We develop an approximation to the first and second moments of a general first-passage time problem in the limit of large, but finite, populations using Kramers–Moyal expansion techniques. We demonstrate these results by application to a stochastic birth-death model for a population of cells in order to develop several approximations to the normal tissue complication probability (NTCP): a problem arising in the radiation treatment of cancers. We specifically allow for interaction between cells, via a nonlinear logistic growth model, and our approximations capture the effects of intrinsic noise on NTCP. We consider examples of NTCP in both a simple model of normal cells and in a model of normal and damaged cells. Our analytical approximation of NTCP could help optimise radiotherapy planning, for example by estimating the probability of complication-free tumour under different treatment protocols.

## Introduction

The first-passage time problem of a general stochastic birth-death process involves finding the distribution of times for which the random process first passes through a specific threshold. Here, we derive an approximation to the first and second moments of a general first-passage time problem. We ‘lead by example’ and describe our results in the context of calculating normal tissue complication probabilities: a problem arising in the radiation treatment of cancer tumours. However, since the linear-noise approximation (LNA), on which our approach is based, is ubiquitous in statistical physics and applications, our result also lends itself to applications in other fields outside of radiotherapy^1^.

Our approximation involves first writing the master equation of the birth-death process, and subsequently approximating the dynamics by carrying out a Kramers–Moyal expansion and LNA. We then proceed to approximate the first-passage time by considering the dynamics in a small region near the boundary. This provides a Gaussian approximation of the first-passage times. While our approximation is relatively crude, the mathematical simplicity of our result is a strength. In some examples closed-form expressions can be obtained. In other cases a small set of ordinary differential equations (ODEs) needs to be solved numerically, which can be done much more efficiently than integrating forward a potentially high-dimensional master equation.

There are many applications which depend on knowing the first-passage time through a boundary. We focus here on treating a cancerous tumour with radiation; in this situation it is likely that the surrounding healthy tissue will also be damaged. Therefore, a radiotherapy treatment protocol aims to provide enough radiation to the tumour to control the cancer whilst not causing excessive side-effects by damaging surrounding tissue. To this end, a protocol must find a balance between maximising the tumour control probability (TCP) and minimising the normal tissue complication probability (NTCP). Normal tissue complications (NTCs) encompass a wide variety of problems ranging in severity from increased urinary frequency from the treatment of prostate cancers^2,3^, to severe neurological complications such as myelitis from the treatment of neck cancers^4^ and organ failure^2^.

There are numerous models of TCP and NTCP in the literature. Broadly, the term ‘model’ is used to describe two different types of mathematical approaches to characterising these probabilities; statistical models and mechanistic models. We concentrate on the second type, which seeks to compute TCP and NTCP ‘bottom-up’ from mechanistic principles of the population dynamics of tumour or normal cells^5,6^. These models are often stylised, but their mutual key characteristic is that they describe the dynamics of cell division and death. Many of these models are intrinsically stochastic. Mitosis and cell death are random events in such models, and the precise outcome is therefore uncertain; the tumour may or may not be controlled, and NTCs can arise, but do not have to. The aim of this line of research is to obtain, for a given model of the population dynamics of cells and a given radiation protocol, the TCP and NTCP. The word ‘obtain’ includes by computer simulation of the population, or by direct mathematical computation when this is possible. While simulations are sometimes viable, the mathematical route when available is generally preferable as explicit formulae provide an efficient way of evaluating TCP or NTCP, often much faster than simulation. Not all types of population dynamics can be treated mathematically exactly however. In such cases approximations have to be made in the mathematical calculation of TCP and NTCP.

TCP computed as the first-passage distribution of a stochastic birth-death model has previously been described by Zaider and Minerbo^6^, and extended in subsequent work^7–9^, using generating-function methods. The use of such methods to solve first-passage time problems, however, is limited to those considering the extinction of all cells and where the dynamics are linear, and so are not directly applicable to NTCP.

A stochastic birth-death model of normal tissue cells where cell death rates are affected by the dose and timing of radiotherapy was described by Stocks *et al*.^10^. NTCP can be seen as the cumulative distribution function of the first-passage time of this stochastic birth-death process through a boundary^10^; NTC sets in when the number of functional cells falls below a certain threshold. However, the analysis by Stocks *et al*.^10^ of this model was restricted to the deterministic limit in which intrinsic noise within the population is not taken into account, and therefore NTCP was approximated as either zero or one.

The application of our theoretical results in approximating the distribution of first-passage times extends the analysis in Stocks *et al*.^10^ to capture features of intrinsic noise. Note that we do not aim to develop a novel model of NTCP, but instead further the analysis of existing models^5,10^ using novel mathematical developments while maintaining the assumptions, and therefore clinical relevance, of previous work.

Mathematically, our main result is intuitive. We find that, for a sufficiently large population, the distribution of first-passage times through a threshold is approximately normal. The variance of this normal distribution decreases proportionally to the size of the population. The deterministic result for NTCP by Stocks *et al*.^10^ is recovered in the limit of infinite population size.

The remainder of this paper is set out as follows. We first present the microscopic model of normal tissue cells adapted from the model of Stocks *et al*.^10^ and a definition of NTCP. We use this model to explain the steps of our approximation and derive our main results for approximating the first-passage time of a birth-death process through a boundary. Following Hanin and Zaider^5^ we then consider a more complicated model of two cell types (normal and ‘doomed’), and we show how our method can be extended to systems with more than one degree of freedom. In the context of this model we also develop a second approximation method for NTCP.

## Logistic model of healthy tissue

### Model definitions

We first focus on a model of normal tissue, similar to that in Stocks *et al*.^10^, which we refer to as Model 1. The model describes a well-mixed population of identical and independent cells; we write *N*_*t*_ for the size of the population at time *t*. The model does not distinguish between organ stem cells, functional cells or different types of tissue renewal but simply assumes that all cells can divide by mitosis with the same rate. We assume that overall growth is limited by spatial constraints, the presence of nutrients, or other regulatory mechanisms. For a population of size *N*, the overall growth rate *b*_*N*_ is a logistic function,

1 $${b}_{N}=\{\begin{array}{cc}b\left(1,-,\frac{N}{K}\right) & {\rm{i}}{\rm{f}}\,N\le K\\ 0 & {\rm{o}}{\rm{t}}{\rm{h}}{\rm{e}}{\rm{r}}{\rm{w}}{\rm{i}}{\rm{s}}{\rm{e}},\end{array}$$

where *b* > 0 is a constant parameter. This indicates that the per capita birth rate decreases with increasing population size, and growth ceases completely when the carrying capacity *K* is reached; *K* is a model parameter and constant in time.

Cells can die due to natural causes and from external radiation. Natural death occurs with rate *d*. We note that explicitly separating death processes from birth events is necessary for a stochastic treatment of the model; basing the analysis on an effective net growth rate (i.e., *b*_*N*_ − *d*) is insufficient to model the dynamics outside of the deterministic limit. External radiation damage to cells is captured via a hazard function *h*(*t*), denoting the per capita death rate due to radiation at time *t*^10,11^. This rate will generally depend on time, as determined by the details of the specific treatment protocol applied and biological assumptions made.

Model 1 can be summarised as a list of ‘reactions’, with notation similar to that used in chemical reaction systems, where we write ${\mathscr{N}}$ to represent an individual normal cell

2 $$\begin{array}{lll}{\mathscr{N}}\mathop{\longrightarrow }\limits^{b\left(1-\frac{N}{K}\right)} & {\mathscr{N}}+{\mathscr{N}} & ({\rm{mitosis}}),\\ {\mathscr{N}}\mathop{\longrightarrow }\limits^{d} & \varnothing  & ({\rm{natural}}\,{\rm{death}}),\\ {\mathscr{N}}\mathop{\longrightarrow }\limits^{h(t)} & \varnothing  & ({\rm{death}}\,{\rm{due}}\,{\rm{to}}\,{\rm{radiation}}),\end{array}$$

where the rates above the arrows are per capita rates.

The deterministic rate equation for this system can be formulated heuristically as follows,

3 $$\frac{{\rm{d}}N}{{\rm{d}}t}=bN\left(1-\frac{N}{K}\right)-[d+h(t)]N.$$

It can also be derived systematically from the lowest-order terms in an expansion in the inverse system size, as discussed below.

In the absence of radiation [i.e., when *h*(*t*) = 0], the non-zero fixed point of Eq. (3) is given by ${N}^{\ast }=K(1-\frac{d}{b})$, where we have assumed *b* > *d*. Since the population dynamics are stochastic, in the absence of radiation the size of the population fluctuates about this value. To simplify the notation we will use $K=\frac{M}{1-\mathrm{d/b}}$ in the following, such that—in the absence of radiation—the average population size is *M*.

### Master equation

The process defined by Eq. (2) can equivalently be described by a (chemical) master equation^12^, where we write *P*_*N*_(*t*) for the probability that the population has size *N* at time *t*

4 $$\begin{array}{cc}\frac{{\rm{d}}}{{\rm{d}}t}{P}_{N}(t)= & (N-1)b\left(1-\frac{N-1}{K}\right){P}_{N-1}(t)+(N+1)[d+h(t)]{P}_{N+1}(t)\\  & -\,Nb\left(1-\frac{N}{K}\right){P}_{N}(t)-N[d+h(t)]{P}_{N}(t),\end{array}$$

or equivalently

5 $$\frac{{\rm{d}}}{{\rm{d}}t}{P}_{N}(t)=({{\mathscr{E}}}^{-1}-1)[Nb\left(1-\frac{N}{K}\right){P}_{N}(t)]+({\mathscr{E}}-1)[N(d+h(t)){P}_{N}(t)],$$

for all *N* < *K*, where ${\mathscr{E}}$ is the step operator defined by its effect on a function *f*_*N*_, i.e., we have ${\mathscr{E}}[{f}_{N}]={f}_{N+1}$, and similarly ${{\mathscr{E}}}^{-1}[{f}_{N}]={f}_{N-1}$.

### Definition of normal-tissue complication probability and strategies to calculate it

An organ requires a minimum number of cells to function properly^13^. We introduce a threshold *L* and say that a normal tissue complication (NTC) is encountered when the number of cells in the population *N*_*t*_ falls below *L*. Given that *N*_*t*_ is a stochastic process, NTC will occur at different times in different realisations of the model dynamics (or potentially, it may never occur in a given realisation). This leads to the definition of normal tissue complication probability (NTCP). We assume that once NTC has been encountered in a given realisation of the dynamics, it cannot be repaired, even if the number of cells ultimately recovers to values above the threshold *L*. We therefore define NTCP(*t*) as the probability that, at some time before *t*, the population contained *L* cells or fewer. Mathematically the calculation of NTCP constitutes a first-passage time problem^14^. More precisely, NTCP(*t*) is the cumulative distribution function of the first-passage time through the threshold *L*.

Realisations of the process defined by Eq. (2) can be generated using the stochastic simulation algorithm by Gillespie^15,16^. In principle, a large ensemble of such simulations can be used to measure NTCP(*t*). However, in practice this approach is of limited use since a large number of runs need to be collected to obtain sufficient statistics. Simulations also offer relatively little in the way of mechanistic insight.

One can also find the NTCP(*t*) by direct numerical integration of Eq. (5) (subject to an absorbing boundary at *L*). In practice, this approach is computationally costly, especially in more realistic models where there are several different types of cells and the master equation is then a large set of coupled ODEs.

An alternative approach involves the use of generating functions^12^. However, this technique is usually only viable for relatively simple models (and not for the above logistic growth process). For example, generating functions can be calculated analytically when per capita birth and death rates do not depend on the current population size, i.e., when *b*_*N*_ is independent of *N*. A notable example of an exact calculation using generating functions is the work of Zaider and Minerbo^6^ who obtain TCP in closed form for a linear-birth death process with time-dependent death rate due to irradiation.

Given the limitations of these numerical and analytical methods, we now develop and use an approximation to estimate NTCP. The approach is based on Kramers–Moyal expansion techniques^12,17^ and retains features of the intrinsic noise resulting from the finiteness of the population of cells. At the same time, we assume that the population is sufficiently large so that the jump process defined by the master Eq. (5) can be approximated by a stochastic differential equation (SDE).

### Kramers–Moyal expansion and linear-noise approximation

The expansion method is based on the assumption of a large, but finite population. We refer to *M* as the average system size and introduce the population density *n*_*t*_ = *N*_*t*_/*M*^12,17^. We re-scale the threshold for the onset of NTC in the same way $\ell =L/M$; NTC thus occurs when ${n}_{t}\le \ell$. We also introduce a re-scaled carrying capacity *k* = *K*/*M*. Given our above choice $K=\frac{M}{1-d/b}$, we have $k={(1-d/b)}^{-1}$.

Re-writing functions of *N* as functions of *n* = *N*/*M*, we find ${{\mathscr{E}}}^{\pm 1}f(n)=f(n\pm 1/M)$ for the action of the step operator. We proceed to consider the limit where the system size is large, *M* ≫ 1. In this limit one can expand

6 $${{\mathscr{E}}}^{\pm 1}=1\pm \frac{1}{M}\frac{\partial }{\partial n}+\frac{1}{2{M}^{2}}\frac{{\partial }^{2}}{\partial {n}^{2}}+\ldots .$$

Substituting this into the master Eq. (5) results in a Fokker–Planck equation for the probability density ∏(*n*, *t*),

7 $$\frac{\partial }{\partial t}\Pi (n,t)=-\frac{\partial }{\partial n}[\mu (n,t)\Pi (n,t)]+\frac{1}{2M}\frac{{\partial }^{2}}{\partial {n}^{2}}[{\sigma }^{2}(n,t)\Pi (n,t)],$$

where we have neglected higher-order terms in *M*^−1^. The probability of finding the random process *n*_*t*_ with a value in the interval $[n,n+{\rm{d}}n)$ at time *t* is ∏(*n*, *t*)d*n*.

For the current model, the drift and diffusion terms in Eq. (7) are given by

8a $$\mu (n,t)=nb\left(1-\frac{n}{k}\right)-n[d+h(t)],$$

8b $${\sigma }^{2}(n,t)=nb\left(1-\frac{n}{k}\right)+n[d+h(t)],$$

respectively. Equation (7) describes the statistics generated by solutions of the It $\bar{{\rm{o}}}$ SDE

9 $${\rm{d}}{n}_{t}=\mu ({n}_{t},t){\rm{d}}t+{M}^{-1/2}\sigma ({n}_{t},t){\rm{d}}{W}_{t},$$

where *W*_*t*_ is a standard Wiener process.

In principle, trajectories of this SDE can be generated in simulations, for example using the Euler–Maruyama method^18^. These simulations are more efficient than simulating the original model, in particular the population size only enters in the noise strength and does not affect computing time required to generate a set number of realisations. However, our aim is to make analytical progress. This requires further approximation, first because *μ*(*n*_*t*_, *t*) is a non-linear function of *n*_*t*_, and more importantly because the noise in Eq. (9) is multiplicative. We proceed by making a further simplification using the LNA^12,17^, effectively turning multiplicative noise into additive noise.

To carry out the LNA we introduce the stochastic process *ξ*_*t*_ via the transformation^17^

10 $${n}_{t}=\phi (t)+{M}^{-1/2}{\xi }_{t},$$

where *ϕ*(*t*) is a deterministic function of *t*, to be determined shortly.

We next substitute this ansatz into Eq. (9) and expand in powers of *M*^−1/2^. From the two lowest-order terms we find

11a $$\frac{{\rm{d}}\phi }{{\rm{d}}t}=\mu [\phi (t),t],$$

11b $${\rm{d}}{\xi }_{t}=\mu {\prime} [\phi (t),t]{\xi }_{t}{\rm{d}}t+\sigma [\phi (t),t]{\rm{d}}{W}_{t},$$

where $\mu {\prime} [\phi (t),t]$ is the partial derivative of the drift *μ*(*n*, *t*) with respect to *n*, evaluated at *ϕ*(*t*) and *t*.

The first of these equations indicates that *ϕ*(*t*) is the solution of a deterministic rate equation in the limit *M* → ∞. Within the linear-noise approximation, the population density *n*_*t*_ is normally distributed with mean *ϕ*(*t*). Up to re-scaling of *N* and *K* this rate equation is identical to Eq. (3). The SDE (11b) describes fluctuations about this deterministic trajectory, due to demographic noise. We note that the LNA is only valid provided corrections to the deterministic dynamics remain small; if this is not the case higher-order terms in the system-size expansion become important. The approximation is generally appropriate if the deterministic trajectory is locally attracting, i.e., if $\mu {\prime} [\phi (t),t] < 0$ at all times. This condition is fulfilled in the present model.

The linear SDE (11b) can be solved straightforwardly^12,17,19^, and, within the LNA, the distribution of *n*_*t*_ is found to be Gaussian, centred around the solution *ϕ*(*t*) of Eq. (11a),

12 $$\Pi (n,t)=\frac{1}{\sqrt{2\pi {M}^{-1}{\Sigma }^{2}(t)}}\exp \left(-\frac{{[n-\phi (t)]}^{2}}{2{M}^{-1}{\Sigma }^{2}(t)}\right).$$

The variance of this distribution, *M*^−1^∑^2^(*t*), is a function of time, and can be obtained from the solution of

13 $$\frac{{\rm{d}}{\Sigma }^{2}}{{\rm{d}}t}=2\mu {\prime} [\phi (t),t]{\Sigma }^{2}(t)+{\sigma }^{2}[\phi (t),t],$$

see e.g. Risken and Frank^19^.

For some cases Eqs. (11a) and (13) can be solved exactly, and one can obtain an analytical expression for ∏(*n*, *t*) in Eq. (12). We discuss this in the context of the current model below. For the general case, these equations can be integrated forward numerically, using standard Runge–Kutta methods. This only requires the integration of two ODEs.

### Approximation of NTCP(*t*)

We now proceed to estimate NTCP using the outcome of the LNA. Taking Eqs. (11a) and (11b) as a starting point, the calculation of NTCP amounts to a first-passage time problem for an SDE with time-dependent drift and noise strength. Equation (11b) describes an Ornstein–Uhlenbeck process with time-dependent rates^12^. Due to the time-dependence of *ϕ*(*t*) in Eq. (11a), calculating NTCP amounts to calculating the first-passage time of this Ornstein-Uhlenbeck process through a moving boundary. While the first-passage time distribution of Ornstein–Uhlenbeck processes is available for constant rates and a static boundary^20^, studies of instances with time-dependence are often based on approximation schemes for specific cases; examples can be found in the literature^21,22^.

**Figure 1:**
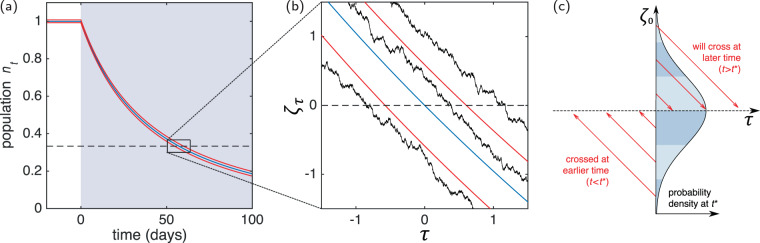
Population size as a function of time for Model 1. In this set-up constant radiation acts from a given time, here chosen to be *t* = 0. The size of the population then decreases and falls below the threshold for the onset of NTCs. (**a**) The central, blue line shows the deterministic trajectory, Eq. (11a), the red lines show a band of one standard deviation as predicted by the LNA, see Eq. (13). The shading of the background indicates the rate of cell death due to radiation *h*(*t*). The dashed line is the threshold for onset of NTC. (**b**) Magnified look at the crossing region, shown in the re-scaled coordinates *τ* and *ζ*. Shown are three stochastic trajectories (black noisy lines) from simulation of SDE (10); they are approximately linear with gradient minus one, as predicted by Eq. (16). (**c**) Schematic representation of our approximation. We start from the Gaussian distribution obtained within the LNA [Eq. (12)] and project trajectories onto the time axis, assuming that their behaviour is linear with slope minus one. Model parameters are given in Table 2 (parameter set (D)).

To make progress we therefore use a further approximation. We focus on cases in which the deterministic trajectory *ϕ*(*t*) crosses the threshold $\ell =L/M$, as illustrated in Fig. 1(a); we write t^*^ for this time. The exact value of t^*^ will depend on the applied radiation protocol and the other model parameters. The calculation of NTCP(*t*) by Stocks *et al*.^10^ is based on this deterministic contribution, and within their calculation NTCP (*t*) = Θ(*t* − t^*^) is a Heaviside step function [Θ(*u*) = 1 for *u* ≥ 0, and Θ(*u*) = 0 otherwise]. Our aim is to build on these results and to capture some of the influence of intrinsic fluctuations on NTCP.

As a next step we look at the dynamics of Eqs. (11a) and (11b) in a time window around t^*^, as shown in Fig. 1(b). Some trajectories of the stochastic system will cross the threshold $\ell$ before t^*^, and others after t^*^. We expect these fluctuations in the crossing time to decrease as the system-size parameter *M* is increased. To evaluate this further we consider the Gaussian distribution for the population density ${n}_{{t}^{\ast }}$ obtained by evaluating Eq. (12) at time t^*^. By construction, this distribution is centred on $\ell$, as shown in Fig. 1(c). We now proceed on the basis that trajectories with values ${n}_{{t}^{\ast }} > \ell$ will first cross the threshold at a time greater than t^*^, and estimate this time of crossing from the dynamics near t^*^. Similarly, trajectories with ${n}_{{t}^{\ast }} > \ell$ have already crossed the threshold, and we estimate how long before t^*^ this has occurred. This procedure implies several assumptions, for example a trajectory with ${n}_{{t}^{\ast }} > \ell$ may have had its first crossing before t^*^ and then returned to values *n*_*t*_ above $\ell$ due to further fluctuations. This is not captured by our estimate of NTCP.

In order to focus on the dynamics in a time window near t^*^, it is useful to introduce re-scaled coordinates

14a $$t={t}^{\ast }-\frac{{M}^{-\mathrm{1/2}}}{\mu (\ell ,{t}^{\ast })}\tau ,$$

14b $${n}_{\tau }=\ell +{M}^{-\mathrm{1/2}}{\zeta }_{\tau }.$$

Considering values of *τ* and *ζ* of order *M*^0^ allows us to magnify the region around t^*^ where boundary crossings are likely (*ζ*_*τ*_ refers to the random process, while *ζ* is a value in the process’s state space). In these coordinates, the crossing of the deterministic trajectory occurs at *τ* = 0, and the position of the threshold is at *ζ* = 0. We note that $\mu (\ell ,{t}^{\ast }) < 0$ so that positive values of the re-scaled time (*τ* > 0) correspond to *t* > t^*^. A summary of the coordinates used in our analysis is given in Table 1.

**Table 1:** Summary of the different coordinate systems used to describe the population in Model 1.

	Coordinate	Interpretation	Relations
(A)	*N*_*t*_	number of individuals in population at time *t*	—
(B)	*n*_*t*_	population density	*n*_*t*_ = *N*_*t*_/*M*
(C)	*ϕ*(*t*)	deterministic (mean-field) trajectory	*n*_*t*_ = *ϕ*(*t*) + *M*^−1/2^*ξ*_*t*_
	*ξ*_*t*_	deviation from mean-field path due to linear noise	
(D)	*ζ*_*τ*_	re-scaled population near boundary $\ell =L/M$	${n}_{\tau }=\ell +{M}^{-\mathrm{1/2}}{\zeta }_{\tau }$
	*τ*	re-scaled time near deterministic crossing time t*	$t={t}^{\ast }+\frac{{M}^{-\mathrm{1/2}}}{-\mu (\ell ,{t}^{\ast })}\tau$

Original coordinates (A) appear in the master Eq. (5), while coordinates (B) and (C) are used in the Kramers–Moyal expansion and linear-noise approximation, respectively [see Eqs. (9) and (11)]. Coordinates (D) are used for our analysis of the dynamics in the narrow, boundary-crossing region. The subscript *t* (or *τ*) is used to denote random processes.

Substituting the new coordinates into Eq. (7), and writing $\tilde{\Pi }(\zeta ,\tau )$ for the probability density in these coordinates, we find

15 $$\begin{array}{cc}\frac{\partial }{\partial \tau }\tilde{\Pi }(\zeta ,\tau )= & \frac{1}{\mu (\ell ,{t}^{\ast })}\frac{\partial }{\partial \zeta }[\mu (\ell +{M}^{-\mathrm{1/2}}\zeta ,t)\tilde{\Pi }(\zeta ,\tau )]\\  & +\,\frac{1}{\mu (\ell ,{t}^{\ast })}\frac{1}{2{M}^{\mathrm{1/2}}}\frac{{\partial }^{2}}{\partial {\zeta }^{2}}[{\sigma }^{2}(\ell +{M}^{-\mathrm{1/2}}\zeta ,t)\tilde{\Pi }(\zeta ,\tau )].\end{array}$$

Expanding in powers of *M*^−1/2^ we find to lowest order $\frac{\partial }{\partial \tau }\Pi (\zeta ,\tau )=\frac{\partial }{\partial \zeta }\Pi (\zeta ,\tau )$, i.e., near the threshold the dynamics of the system can be approximated by

16 $${\zeta }_{\tau }={\zeta }_{0}-\tau ,$$

where *ζ*_0_ is the location of the path at time *τ* = 0 (i.e., at *t* = t^*^). Figure 1(b) shows a number of different stochastic trajectories in this region. Broadly, they travel along approximately parallel straight paths of gradient minus one (in the coordinate system of *τ* and *ζ*).

We now use this result to approximate the distribution of crossing times. To do this we estimate when a particular trajectory located at *ξ*_0_ at time t^*^ crosses (or did cross) the threshold. We write *τ*_×_(*ζ*_0_) for this crossing time in the re-scaled coordinates. Using Eq. (16) we find

17 $${\tau }_{\times }({\zeta }_{0})={\zeta }_{0}.$$

We show this schematically in Fig. 1(c). We now combine this with the Gaussian distribution for *ξ*_0_ obtained from the LNA, also shown in Fig. 1(c). Equation (12), evaluated at *t* = t^*^, can be written as

18 $$\Pi ({\zeta }_{0})=\frac{1}{\sqrt{2\pi {\Sigma }^{2}({t}^{\ast })}}\exp \left(-\frac{{\zeta }_{0}^{2}}{2{\Sigma }^{2}({t}^{\ast })}\right),$$

and we use this together with Eq. (17) to approximate the distribution of first-passage times *t*_×_ as

19 $$p({t}_{\times })=\sqrt{\frac{M{\mu }^{2}(\ell ,{t}^{\ast })}{2\pi {\Sigma }^{2}({t}^{\ast })}}\exp \left(-\frac{M{\mu }^{2}(\ell ,{t}^{\ast })}{2{\Sigma }^{2}({t}^{\ast })}{({t}_{\times }-{t}^{\ast })}^{2}\right).$$

Using the definition of NTCP as outlined above we find

20 $$\,NTCP\,(t)=\frac{1}{2}\left[1+{\rm{erf}}\left(\frac{(t-{t}^{\ast })\sqrt{M}\mu (\ell ,{t}^{\ast })}{\sqrt{2}\Sigma ({t}^{\ast })}\right)\right],$$

where erf is the error function.

We now test this approximation scheme on the logistic growth model defined in Eq. (2). We focus on a particularly simple case where there is no radiation prior to a certain time, and a constant rate of cell death due to radiation thereafter so that the hazard function *h*(*t*) is the step function

21 $$h(t)=\{\begin{array}{cc}0 & {\rm{f}}{\rm{o}}{\rm{r}}\,t < 0,\\ {h}_{0} & {\rm{f}}{\rm{o}}{\rm{r}}\,t\ge 0.\end{array}$$

We primarily consider radiation of this type as a simple initial example, following the study of NTCP in Stocks *et al*.^10^. For this case, analytical and numerical methods exist for obtaining the moments of the first-passage time^23^. More complicated radiation protocols (for which no such methods are available) will be discussed in later examples.

We assume that the dynamics of the population start long before *t* = 0, so that the stationary state of the master Eq. (5) (with *h*(*t*) = 0) is reached by *t* = 0. The mean and variance of this stationary distribution are given by the fixed points of Eqs. (11a) and (13), using *μ* and *σ*^2^ for the logistic model and setting *h*(*t*) = 0. We have

22a $$\phi (t=0)=1,$$

22b $$\Sigma (t=0)=\frac{d}{b-d}.$$

At times *t* ≥ 0, Eqs. (11a) and (13) are given by

23a $$\frac{{\rm{d}}\phi }{{\rm{d}}t}=\phi b\left(1-\frac{\phi }{k}\right)-\phi [d+{h}_{0}],$$

23b $$\frac{d{\Sigma }^{2}}{dt}=2\left\{b\left(1-\frac{2\phi }{k}\right)-[d+{h}_{0}]\right\}{\Sigma }^{2}+\phi b\left(1-\frac{\phi }{k}\right)+\phi [d+{h}_{0}].$$

Equation (23a) can be solved in closed form subject to the initial condition *ϕ*(0) = 1 to give the deterministic trajectory *ϕ*(*t*) as

24 $$\phi (t)=\{\begin{array}{cc}\frac{(b-d-{h}_{0}){e}^{(b-d-{h}_{0})t}}{(b-d)({e}^{(b-d-{h}_{0})t}-1)+(b-d-{h}_{0})} & {\rm{i}}{\rm{f}}\,b-d-{h}_{0}\ne 0\\ \frac{1}{(b-d)t+1} & {\rm{i}}{\rm{f}}\,b-d-{h}_{0}=0.\end{array}$$

From this, one then finds the passage time t^*^ of the deterministic trajectory as

25 $${t}^{\ast }=\frac{1}{b-d-{h}_{0}}\,\log \left(\frac{{h}_{0}\ell }{b\ell -d\ell -b+d+{h}_{0}}\right),$$

for *b − d* − *h*_0_ ≠ 0, assuming the fixed point of the deterministic trajectory is below the boundary $\ell$. Next we turn to Eq. (23b) in order to find ∑^2^(t^*^). For constant radiation the path *ϕ*(*t*) is monotonically decreasing in time. This allows us to trade the time derivative in Eq. (23b) for a derivative with respect to *ϕ*, resulting in a linear ODE for ∑^2^ as a function of *ϕ*. For our specific example this ODE can be solved in closed form, and we find the variance of first-passage times as

26 $$\begin{array}{cc}\frac{{\Sigma }^{2}({t}^{\ast })}{M{\mu }^{2}(\ell ,{t}^{\ast })}= & \frac{5b+\frac{2(b-d)d}{{h}_{0}}+\frac{(b-2d){h}_{0}}{b-d}-\frac{b+d+{h}_{0}}{\ell }+\frac{(b-d)(b-d-{h}_{0})(d+{h}_{0})}{{[d+{h}_{0}+b(\ell -1)-d\ell ]}^{2}}-\frac{(b-d)[b+3(d+{h}_{0})]}{d+{h}_{0}+b(\ell -1)-d\ell }}{M{(b-d-{h}_{0})}^{3}}\\  & +\,\frac{2(b-d)(b+2d+2{h}_{0})\log \left(\frac{{h}_{0}\ell }{b\ell -d\ell -b+d+{h}_{0}}\right)}{M{(b-d-{h}_{0})}^{4}}.\end{array}$$

This can then be used in Eq. (20) to obtain NTCP(*t*).

**Figure 2:**
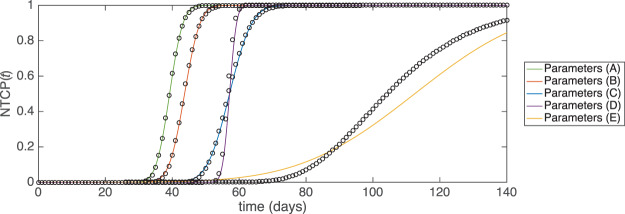
NTCP as a function of time for Model 1. Black circles are obtained from numerical integration of the master equation of the original model, Eq. (5). Coloured solid lines show the approximation of Eqs. (25) and (26). Model parameters are given in Table 2.

In Fig. 2 we show the resulting NTCP as a function of time for several sets of model parameters; these parameter sets are summarised in Table 2, and were previously motivated and used in Stocks *et al*.^10^ to consider normal tissue complications arising from the treatment of prostate cancer. For these sets of parameters, the standard deviation in the time for NTCP onset varies from 3% (parameter set (D)) to 21% (parameter set (E)) of the mean onset time. In order to test the accuracy of our approximation, we have also obtained NTCP(*t*) for the original model by numeral integration of the master equation Eq. (5); these values are shown as black circles in Fig. 2. These results are compared with the analytical approximations in Eqs. (20) and (26), and for most of the parameter sets tested we find good agreement. The approximation works noticeably less well for parameter set (E) than for the other four sets. In this case, the speed with which the deterministic path crosses the boundary is lower than for the other parameter sets. This leads to a longer time window around t^*^ within which crossings are likely, and thus a larger amount of error in our approximation.

**Table 2:** Five sets of parameters used in Fig. 2 for Model 1.

Parameter	Definition	Value				
		(A)	(B)	(C)	(D)	(E)
*b*	mitosis rate (day^−1^)	0.019	0.019	0.019	0.019	0.038
*d*	natural death rate (day^−1^)	0.002	0.002	0.002	0.002	0.004
*h*_0_	irradiated death rate (day^−1^)	0.035	0.032	0.026	0.026	0.026
*M*	typical population size (see text)	500	500	500	5000	500
$\ell$	threshold for onset of NTC	1/3	1/3	1/3	1/3	1/3

These parameter sets are the same as those considered by Stocks *et al*.^10^, but we have defined separate mitosis and natural death rates to be able to analyse stochastic effects in finite populations. The ratio of mitosis and natural death was chosen as 10:1, consistent with literature^5^.

## Extended model of normal and doomed cells

### Model definitions

Hanin and Zaider^5^ proposed a model which adds complexity by including radiation-damaged cells. In this model, damaged cells continue to occupy the limited volume available to the population. Damaged cells also carry out their functions, but fail to proliferate. The presence of such cells has been offered an explanation for the observation that, after irradiation, an initial lag period occurs before re-population^5,24^. We refer to this as Model 2.

As before there are ‘normal cells’ ${\mathscr{N}}$ which carry out the functions of the organ; these cells have the ability to proliferate (and also die with a constant rate *d*_1_ from causes unrelated to radiation). Once damaged by radiation, a normal cell does not vanish immediately; rather, it becomes a ‘doomed cell’ ${\mathscr{X}}$^5^. Doomed cells continue to contribute to the normal functions of the organ, however they are unable to proliferate. Thus, although they may temporarily aid the function of the organ, they ultimately die at a constant rate *d*_2_ without reproducing. Doomed cells also consume resources and so are in direct competition with the normal cells. As a result of this, the per capita mitosis (birth) rate of normal cells decreases as the total size of the population of both types increases. The dynamics of Model 2 can be summarised as follows:

27 $$\begin{array}{lll}{\mathscr{N}}\mathop{\longrightarrow }\limits^{b\left(1-\frac{N+X}{kM}\right)} & {\mathscr{N}}+{\mathscr{N}} & ({\rm{mitosis}}\,{\rm{of}}\,{\rm{normal}}\,{\rm{cells}}),\\ {\mathscr{N}}\mathop{\longrightarrow }\limits^{h(t)} & {\mathscr{X}} & ({\rm{radiation}}\,{\rm{damage}}),\\ {\mathscr{N}}\mathop{\longrightarrow }\limits^{{d}_{1}} & \varnothing  & ({\rm{death}}\,{\rm{of}}\,{\rm{normal}}\,{\rm{cell}}),\\ {\mathscr{X}}\mathop{\longrightarrow }\limits^{{d}_{2}} & \varnothing  & ({\rm{death}}\,{\rm{of}}\,{\rm{doomed}}\,{\rm{cell}}).\end{array}$$

We write *N* and *X* for the numbers of normal and doomed cells, respectively. As before, the constant *k* ≡ (1 − *d*_1_/*b*)^−1^ is chosen so that—in the absence of radiation—the stationary average size of the population of normal cells is *M*. An NTC is assumed to arise when the total number of functional cells, *N* + *X*, falls below a threshold *L*.

Writing *s* = (*N* + *X*)/*M* for the (re-scaled) total number of functional cells in the population, and *x* = *X*/*M* for the (re-scaled) number of doomed cells, one has the following rate equations in the deterministic limit,

28a $$\frac{{\rm{d}}s}{{\rm{d}}t}=b\left(1-\frac{s}{k}\right)(s-x)-{d}_{1}(s-x)-{d}_{2}x,$$

28b $$\frac{{\rm{d}}x}{{\rm{d}}t}=h(t)(s-x)-{d}_{2}x.$$

In this example, as per the literature^5,10^, we consider a hazard function representing brachytherapy where there is a time-varying dose of radiation acting on the population of normal cells, resulting from the decay of a radioactive implant:

29 $$h(t)=\alpha {R}_{0}{e}^{-\lambda t}+\frac{2\beta {R}_{0}^{2}{e}^{-\lambda t}}{\gamma -\lambda }({e}^{-\lambda t}-{e}^{-\gamma t}),$$

where *α*, *β*, *γ*, *λ* and *R*_0_ are model parameters; *R*_0_ in particular denotes the initial dose rate. Further details are given in the Supplementary Information. We consider a specific set of realistic parameters, proposed by Hanin and Zaider^5^ and summarised in Table 3. These parameters were chosen to model the treatment of prostate cancer, where the normal-tissue complication refers to grade 2, or larger, toxicity (‘GU2+’) of the genitourinary tract.

**Table 3:** Parameters used in Fig. 3.

Parameter	Definition	Fig. 3(a,b)	Fig. 3(c,d)
*b*	mitosis rate (day^−1^)	0.0821	0.246
*d*_1_	normal cell death rate (day^−1^)	0.0164	0.0164
*d*_1_	irradiated cell death rate (day^−1^)	0.0164	0.0164
*M*	population size	1000	1000
$\ell =\frac{L}{M}$	critical fraction of population	0.39	0.39
*α*	LQ model parameter (Gy^−1^)	0.109	0.109
*β*	LQ model parameter (Gy^−2^)	0.0364	0.0364
*γ*	rate of DNA repair (day^−1^)	23.7	23.7
*R*_0_	initial dose rate of implant (Gday^−1^)	1.68	1.68
*λ*	decay rate (day^−1^)	0.0117	0.0117

Similar parameters were previously proposed in Hanin and Zaider^5^. We have explicitly included normal-cell birth and death and made the assumption that *d*_1_ = *d*_2_.

### Alternative approximation for NTCP

**Figure 3:**
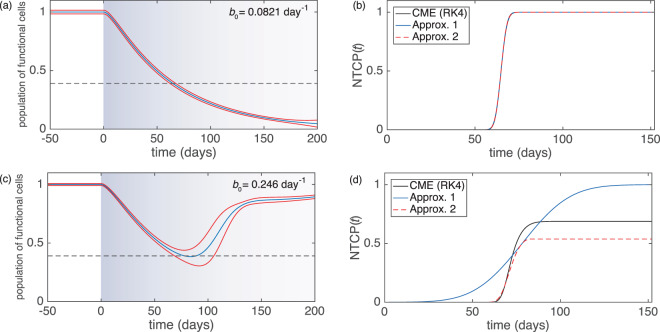
Behaviour of Model 2. (**a**,**c**) Population density for number of functional cells as a function of time for two different parameter sets (see Table 3). The central blue line shows the deterministic trajectory [Eq. (28b)], red lines indicate a band of one standard deviation as predicted by the linear-noise approximation. The shading of the background indicates the rate of radiation damage *h*(*t*). (**b**,**d**) NTCP as a function of time. We compare the results of our two approximations with the outcome of numerical integration of the (chemical) master equation using a Runge–Kutta scheme (RK4).

Results for Model 2 are presented in Fig. 3. We first focus on the deterministic dynamics, indicated by the blue lines in panels (a) and (c). In panel (a) the mitosis rate *b* is sufficiently low for deterministic trajectory to fall below the threshold $\ell$ for the onset of NTCs. The approximation for NTCP developed previously can be applied, as discussed in more detail later.

The second parameter set in Table 3 describes a case with a higher mitosis rate *b*. As shown in Fig. 3(c), the solution of the deterministic rate equations then only briefly falls below the threshold $\ell$. The number of functional cells then increases again to values above $\ell$. In the stochastic system we expect only a fraction of trajectories to cross the threshold; some realisations may never fall below $\ell$, and hence *NTCP*(*t*) can be expected to take a long-time limit below one. This cannot be captured by the approximation method developed previously.

With this in mind, we propose the following improved method of estimating NTCP. Within the LNA, at each moment in time $t$ the distribution of the population of interest (in this case *s*_*t*_) is approximately normal with a mean *ϕ*(*t*) and variance ∑^2^(*t*) given by Eqs. (11a) and (13), respectively. The amount of probability below the threshold $\ell$ at a given time is then obtained as

30 $$Q(t)=\frac{1}{2}\left[1+{\rm{erf}}\left(\frac{\sqrt{M}[\ell -\phi (t)]}{\sqrt{2}\Sigma (t)}\right)\right].$$

We now estimate *NTCP*(*t*) as the maximum amount of probability below the threshold at any earlier time *t* ≤ *t*, i.e.,

31 $${\rm{NTCP}}(t)=\mathop{{\rm{\max }}}\limits_{t{\prime} \le t}\,Q(t{\prime} ).$$

Further steps of the mathematical evaluation are presented in the Supplementary Information. We briefly comment on the limitations of this approximation, before we discuss the results for the model of normal and doomed cells. Equation (31) provides a lower bound for NTCP of the process described by the LNA. This can be seen as follows. At a given time *t*, let the maximum in Eq. (31) have occurred at a time *t*_*m*_ ≤ *t*; the estimate for *NTCP*(*t*) is then *Q*(*t*_*m*_). Consider now a trajectory with a total population density above the boundary at time *n*_*m*_, ${s}_{{t}_{m}} > \ell$. Such a trajectory does not contribute to *NTCP*(*t*) within our approximation, even though the population size might have taken values below the threshold before *t*_m_, or go below threshold between *t*_*m*_ and *t*. The above approximation therefore underestimates NTCP. We note that the SDE obtained in the LNA is itself an approximation, so the above calculation is not necessarily a lower bound to the NTCP of the discrete population dynamics from which we started.

Despite these limitations, the method provides useful estimates for NTCP. For example, *NTCP*(*t*) obtained from Eqs. (30) and (31) for the simple logistic model of healthy tissue does not significantly differ from the predictions of the previous approximation method for *NTCP*(*t*). To keep the language compact we will refer to the previous approximation procedure as Approximation 1 from now on, and to that in Eqs. (30) and (31) as Approximation 2. A quantitative comparison of the distributions of first-passage time from the two approximations for Model 1 is included in the Supplementary Information. It indicates that Approximation 2 provides an improvement relative to Approximation 1 and that both methods do considerably better than the deterministic approximation in Stocks *et al*.^10^.

### NTCP for model of normal and doomed cells

Approximation 2 applied to Model 2 can provide a significantly improved prediction of NTCP compared to Approximation 1, as we will discuss now. In this context it is useful to distinguish the cases in which normal tissue complication occurs with certainty at long times and those in which long-time NTCP stays below one.

#### Certain normal tissue complication at long times

For the first set of parameters in Table 3 normal-tissue complication occurs with probability one at long times. We show results in panel (a) of Fig. 3. The source of radiation is implanted at time zero, assuming that the population of normal cells is at its stationary state at this time. The population of functional cells then decreases monotonously, and the number of functional cells crosses the threshold for the onset of NTC. Panel (b) shows the estimates for *NTCP* as a function of time for Approximation 1 and Approximation 2. Their predictions are largely indistinguishable, and they both agree well with results for the original model found by numerical integration of the master equation.

We note that for this choice of parameter values, carrying out the numerical integration of the master equation takes approximately 10^5^ times longer than to evaluate each of the two approximations. This is because the master equation consists of a set of *M*^2^ coupled ODEs, whereas evaluation of each of the approximations only involves integrating forward five ODEs (for the mean proportion of each type of cell, their variances and the covariance). Thus, the approximation methods offer a significant increase in efficiency for large populations, at moderate reduction of accuracy.

#### Uncertain onset of normal tissue complication

In panels (c) and (d) of Fig. 3 we show the same quantities, but for a different choice of birth rate (see Table 3). The deterministic path barely crosses the boundary $\ell$, and for this choice of parameters only a fraction of trajectories of the stochastic model will lead to an onset of NTC. In this case, the predictions of the two approximations are widely different. Approximation 1 assumes a Gaussian distribution of first-passage times and deviates significantly from the NTCP seen in the original model. Most notably, this approximation predicts that all trajectories eventually cross the boundary so that *NTCP*(*t*) → 1 at large times. Although this is not the case for typical population size used in this example (*M* = 1000), we remark that for *M* → ∞ NTC becomes certain at long times in the original model for the present parameter set.

As seen in Fig. 3(d) Approximation 2 outperforms Approximation 1. This is because, in the narrow region where boundary-crossings are likely, there is a significant change in the drift for the total population size; the sign of the drift changes from negative to positive. Approximation 2 takes this into account, whereas Approximation 1 is based on constant drift within the region near the boundary $\ell$. Unlike Approximation 1, Approximation 2 does not (wrongly) predict that all trajectories eventually cross the boundary. Instead *NTCP*(*t*) remains below unity at *t* → ∞ within Approximation 2.

## Discussion

We have derived approximations for the distribution of the first-passage time of a stochastic birth-death model through a boundary. These approximations capture effects of fluctuations in the population which were discarded in previous approaches. The improvements rely on an expansion in the inverse typical size of the population. One can therefore expect the approach to be particularly useful for large, but finite populations. Intrinsic noise is then weak, but not always weak enough to be ignored altogether. The methods we have developed do not require the birth-death model to be linear, for example we have considered logistic growth.

Our analysis was presented in the context of normal tissue complication probabilities for radiotherapy treatment. In particular, we have obtained approximations of NTCP for models of normal tissue with a single type of cell and for an extended model with two different cell types. Our results demonstrate that these approximations can lead to a significant increase in efficiency over simulation methods, at a moderate loss of accuracy. This is the case particularly when the underlying model becomes complex and has many different internal states. We note that NTCP takes the form of an error function in our approximation; this functional form has previously been reported in statistical models of NTCP, see for example Lyman^25^.

Our applied analysis is limited to stylised models, and we do not claim direct clinical applicability. For example, we assume that NTC occurs when the number of functioning cells first falls below a threshold. This threshold assumption, and more broadly the assumption of a ‘functional reserve’ or critical number of structural elements that must be undamaged to avoid tissue failure, has been widely made in models of NTCP in the literature^5,10,26,27^. We acknowledge that this assumption is more valid for certain types of tissue and complications than others, in particular for tissues arranged ‘in parallel’ rather than ‘in series’^28^. However, to what extent this established assumption is clinically appropriate remains a matter for further biological exploration and is beyond the scope of the mathematical approach of this manuscript. In addition, only a limited number of parameter sets have been investigated in this study and, although informed by previous literature, we note that these serve as an illustration of our methodological developments rather than claiming they are necessarily clinically realistic values.

However, the analytical approaches developed here have the potential to give clinical benefit as more realistic models of cancerous cells and normal tissue evolve. For example, combining approximations of NTCP with values for tumour control probabilities – the probability of eliminating all cancer cells – can give an estimate of the success of a particular treatment protocol. In particular our approximations of NTCP, used with approximations of TCP from the literature, can be used to estimate the probability of complication-free tumour control. This can be used for the efficient identification of optimised parameters for treatment planning; we give more details on this application of our approximations in the Supplementary Information.

One may ask whether the inclusion of intrinsic noise is necessary in modelling NTCP. Hanin and Zaider^5^ argue that deterministic approaches might be sufficient, due to the high numbers of cells involved. However we note that the size of the population may vary depending on context. For example, the model could describe a functional subunit (FSU) of an organ, rather than the entire organ^27,29,30^. NTCP would then not necessarily indicate the probability that an organ fails, but instead that such a subunit no longer fulfils its function. For instance, Niemierko and Goitein consider a kidney split into 10^7^ FSUs, where each FSU contains 10^4^ cells^27^. In such circumstances noise in the population (i.e., within a FSU) may become relevant. Intrinsic stochasticity may also be important in the context of stem cells, especially if they are present in relatively small numbers^31–34^.

To summarise, we have developed approximations to the first and second moment of a general first-passage time problem and applied them to models of cells damaged by radiotherapy. We hope that our mathematical results can be adapted to more clinically realistic models of cancerous cells and normal tissue as they evolve and note that they have wider applicability to other problems, in many wide-ranging fields, in which first-passage times of stochastic processes are of interest.

## Supplementary information

Supplementary Information.

## References

[1] Ralf, M., Sidney, R. & Gleb, O. *First-passage phenomena and their applications*, vol. 35 (World Scientific, 2014).

[2] Martinez E (2015). Permanent seed brachytherapy for clinically localized prostate cancer: Long-term outcomes in a 700 patient cohort. Brachytherapy.

[3] Tanaka N, Asakawa I, Hasegawa M, Fujimoto K (2015). Urethral toxicity after ldr brachytherapy: experience in japan. Brachytherapy.

[4] Horiot J-C (1997). Accelerated fractionation (af) compared to conventional fractionation (cf) improves loco-regional control in the radiotherapy of advanced head and neck cancers: results of the eortc 22851 randomized trial. Radiotherapy and Oncology.

[5] Hanin L, Zaider M (2013). A mechanistic description of radiation-induced damage to normal tissue and its healing kinetics. Physics in Medicine & Biology.

[6] Zaider M, Minerbo G (2000). Tumour control probability: a formulation applicable to any temporal protocol of dose delivery. Physics in Medicine & Biology.

[7] Dawson A, Hillen T (2006). Derivation of the tumour control probability (tcp) from a cell cycle model. Computational and Mathematical Methods in Medicine.

[8] Maler A, Lutscher F (2009). Cell-cycle times and the tumour control probability. Mathematical medicine and biology: a journal of the IMA.

[9] Hillen T, De VrIeS G, Gong J, Finlay C (2010). From cell population models to tumor control probability: including cell cycle effects. Acta Oncologica.

[10] Stocks T, Hillen T, Gong J, Burger M (2016). A stochastic model for the normal tissue complication probability (ntcp) and applications. Mathematical medicine and biology: a journal of the IMA.

[11] Gong J, Dos Santos MM, Finlay C, Hillen T (2011). Are more complicated tumour control probability models better?. Mathematical medicine and biology: a journal of the IMA.

[12] Gardiner, C. W. *et al*. *Handbook of stochastic methods*, vol. 3 (springer Berlin, 1985).

[13] Bond, V. P., Fliedner, T. M. & Archambeau, J. O. *Mammalian radiation lethality: a disturbance in cellular kinetics* (Academic Press, 1965).

[14] Redner, S. *A guide to first-passage processes* (Cambridge University Press, 2001).

[15] Gillespie DT (1976). A general method for numerically simulating the stochastic time evolution of coupled chemical reactions. Journal of computational physics.

[16] Gillespie DT (1977). Exact stochastic simulation of coupled chemical reactions. The journal of physical chemistry.

[17] Van Kampen, N. G. *Stochastic processes in physics and chemistry*, vol. 1 (Elsevier, 1992).

[18] Kloeden, P. E. & Platen, E. *Numerical solution of stochastic differential equations*, vol. 23 (Springer Science & Business Media, 2013).

[19] Risken, H. & Frank, T. *The Fokker-Planck Equation: Methods of Solution and Applications* (Springer-Verlag Berlin Heidelberg, 1996).

[20] Ricciardi LM, Sato S (1988). First-passage-time density and moments of the ornstein-uhlenbeck process. Journal of Applied Probability.

[21] Madec Y, Japhet C (2004). First passage time problem for a drifted ornstein–uhlenbeck process. Mathematical biosciences.

[22] Lo C-F, Hui C-H (2006). Computing the first passage time density of a time-dependent ornstein–uhlenbeck process to a moving boundary. Applied mathematics letters.

[23] D’Onofrio G, Tamborrino M, Lansky P (2018). The jacobi diffusion process as a neuronal model. Chaos: An Interdisciplinary Journal of Nonlinear Science.

[24] Hall, E. J. & Giaccia, A. J. *Radiobiology for the Radiologist*, vol. 6 (Lippincott Williams & Wilkins, 2006).

[25] Lyman JT (1985). Complication probability as assessed from dose-volume histograms. Radiation Research.

[26] Jackson A (1995). Analysis of clinical complication data for radiation hepatitis using a parallel architecture model. International Journal of Radiation Oncology* Biology* Physics.

[27] Niemierko A, Goitein M (1993). Modeling of normal tissue response to radiation: the critical volume model. International Journal of Radiation Oncology* Biology* Physics.

[28] Marks LB (2010). Use of normal tissue complication probability models in the clinic. International Journal of Radiation Oncology* Biology* Physics.

[29] Stavrev P, Stavreva N, Niemierko A, Goitein M (2001). Generalization of a model of tissue response to radiation based on the idea of functional subunits and binomial statistics. Physics in Medicine & Biology.

[30] Tucker SL (2006). Cluster model analysis of late rectal bleeding after imrt of prostate cancer: a case–control study. International Journal of Radiation Oncology* Biology* Physics.

[31] Rutkowska E, Baker C, Nahum A (2010). Mechanistic simulation of normal-tissue damage in radiotherapy–implications for dose–volume analyses. Physics in Medicine & Biology.

[32] D’Andrea, M., Benassi, M. & Strigari, L. Modeling radiotherapy induced normal tissue complications: An overview beyond phenomenological models. *Computational and mathematical methods in medicine* **2016** (2016). 10.1155/2016/2796186 PMC5156873 28044088

[33] Hendry J, Thames H (1986). The tissue-rescuing unit. The British journal of radiology.

[34] Konings AW, Coppes RP, Vissink A (2005). On the mechanism of salivary gland radiosensitivity. International Journal of Radiation Oncology* Biology* Physics.

