# Supplementary material for: First-passage times and normal tissue complication probabilities in the limit of large populations
Source: Sci Rep. 2020 May 29;10:8786. doi: 10.1038/s41598-020-64618-9 (PMC7260376; doi:10.1038/s41598-020-64618-9)
Supplement: Supplementary file 1 — Supplementary Information. [file 41598_2020_64618_MOESM1_ESM.pdf]

Supplementary Information to *First-passage times and normal tissue complication probabilities in the limit of large populations* by Peter G. Hufton, Elizabeth Buckingham-Jeffery and Tobias Galla.

A hazard function representing brachytherapy: the linear-quadratic formalism

We briefly review here the linear-quadratic (LQ) formalism for a radioactive implant[1–3]. We first consider the reaction describing death due to irradiation. The LQ formalism relates the mean surviving fraction of cells ψ to the total dose delivered in a time interval $[0,t]$, $D(t)$:

$$\psi(t) = e^{-\alpha D(t) - \beta q(t) D(t)^2}. \tag{SI.1}$$

Here, there are two radiosensitivity parameters, α and β, which describe a tissue's linear and quadratic responses to a source of radiation, respectively. For a radioactive source exponentially decaying with rate λ and with an initial dose rate R_0, the total dose delivered by time t is given by $D(t) = R_0/\lambda\,[1-\exp(-\lambda t)]$. The function $q(t)$ in Eq. (SI.1) is the Lea–Catcheside protraction factor[4], which is specific to the method of treatment involved. In the case of brachytherapy it is given by

$$q(t) = \frac{2(\lambda t)^2}{(\gamma t)^2(1-\lambda^2/\gamma^2)\left(1-e^{-\lambda t}\right)^2}\left[e^{-(\lambda+\gamma)t} + \gamma t\left(\frac{1-e^{-2\lambda t}}{2\lambda t}\right) - \frac{1+e^{-2\lambda t}}{2}\right]. \tag{SI.2}$$

Here, γ is the rate at which radiation-damaged cells repair their DNA. The fractional change in the population over an infinitesimal time $\dot{\psi}(t)/\psi(t)$ gives the hazard function $h(t)$. This is found to be given by[5]

$$h(t) = \alpha R_0 e^{-\lambda t} + \frac{2\beta R_0^2 e^{-\lambda t}}{\gamma - \lambda}\left(e^{-\lambda t} - e^{-\gamma t}\right). \tag{SI.3}$$

Evaluation of Approximation 1 for Model 2

In Model 2 we write N_t for the number of normal cells at time t and X_t for the number of doomed cells. We are interested in the population of total functional cells, $S_t \equiv N_t + X_t$. Specifically, we are interested in the time S_t first passes a boundary L. The master equation can be formulated in terms of S and X:

$$\begin{aligned}\frac{\mathrm{d}}{\mathrm{d}t}P_{S,X}(t) = {} & \left(\mathscr{E}_S^{-1}-1\right)\left[b(S-X)\left(1-\frac{S}{kM}\right)P_{S,X}(t)\right] \\ & + \left(\mathscr{E}_X^{-1}-1\right)\left[h(t)(S-X)P_{S,X}(t)\right] \\ & + \left(\mathscr{E}_S^{+1}-1\right)\left[d_1(S-X)P_{S,X}(t)\right] \\ & + \left(\mathscr{E}_S^{+1}\mathscr{E}_X^{+1}-1\right)\left[d_2 X P_{S,X}(t)\right],\end{aligned} \tag{SI.4}$$

where $P_{S,X}(t)$ is the probability that random processes S_t, X_t have the values S, X at time t. The operator $\mathscr{E}_S$ is the step operator affecting the size of the total population, and $\mathscr{E}_X$ is the step operator affecting the number of doomed cells, i.e. $\mathscr{E}_S[f_{S,X}] = f_{S+1,X}$ and $\mathscr{E}_X[f_{S,X}] = f_{S,X+1}$.

We proceed by approximating the master equation via a Kramers–Moyal expansion. First, we introduce re-scaled processes $s_t = S_t/M$ and $x_t = X_t/M$, and then expand the step operators in the limit $M \gg 1$. We arrive at the Fokker–Planck equation

$$\begin{aligned}\frac{\partial}{\partial t}\Pi(s,x,t) = {} & -\frac{\partial}{\partial s}\left[b\left(1-\tfrac{s}{k}\right)(s-x) - d_1(s-x) - d_2 x\right]\Pi(s,x,t) \\ & - \frac{\partial}{\partial x}\left[h(t)(s-x) - d_2 x\right]\Pi(s,x,t) \\ & + \frac{1}{2M}\frac{\partial^2}{\partial s^2}\left[b(1-s)(s-x) + d_1(s-x) + d_2 x\right]\Pi(s,x,t) \\ & + \frac{1}{2M}\frac{\partial^2}{\partial x^2}\left[h(t)(s-x) + d_2 x\right]\Pi(s,x,t) \\ & + \frac{1}{M}\frac{\partial}{\partial s}\frac{\partial}{\partial x}\, d_2 x \Pi(s,x,t),\end{aligned} \tag{SI.5}$$

where we have neglected higher-order terms in M^{-1}. This Fokker–Planck equation can equivalently be written as an SDE:

$$\begin{pmatrix} \mathrm{d}s_t \\ \mathrm{d}x_t \end{pmatrix} = \vec{\mu}(s_t, x_t)\mathrm{d}t + \frac{1}{M^{1/2}}\mathbf{B}(s,x,t)\begin{pmatrix} \mathrm{d}W_t^{(1)} \\ \mathrm{d}W_t^{(2)} \end{pmatrix}, \tag{SI.6}$$

where the drift is given by

$$\vec{\mu}(s,x) = \begin{pmatrix} b\left(1-\frac{s}{k}\right)(s-x) - d_1(s-x) - d_2 x \\ h(t)(s-x) - d_2 x \end{pmatrix}. \tag{SI.7}$$

The diffusion $\mathbf{B}(s,x,t)$ is the positive-semidefinite matrix satisfying

$$\mathbf{B}^2(s,x,t) = \begin{pmatrix} b\left(1-\frac{s}{k}\right)(s-x) + d_1(s-x) + d_2 x & d_2 x \\ d_2 x & h(t)(s-x) + d_2 x \end{pmatrix}. \tag{SI.8}$$

We proceed by linearising the SDE (SI.6). Let $s_t = \phi_1(t) + M^{-1/2}\xi_{1_t}$ and $x_t = \phi_2(t) + M^{-1/2}\xi_{2_t}$, where $\phi_1(t)$ and $\phi_2(t)$ are the deterministic functions of time. Substituting and collecting lowest order terms, we see these functions are given by the ODEs

$$\frac{\mathrm{d}\phi_1}{\mathrm{d}t} = \left(1 - \frac{\phi_1}{k}\right) b(\phi_1 - \phi_2) - d_1(\phi_1 - \phi_2) - d_2\phi_2, \tag{SI.9a}$$

$$\frac{\mathrm{d}\phi_2}{\mathrm{d}t} = h(t)(\phi_1 - \phi_2) - d_2\phi_2, \tag{SI.9b}$$

i.e., we recover Eqs. (28b).

The random processes ξ_{1_t} and ξ_{2_t} describe deviations from this deterministic trajectory, and are of the Ornstein–Uhlenbeck type

$$\mathrm{d}\vec{\xi}_t = \mathbf{A}(\phi_1, \phi_2, t)\,\vec{\xi}_t \mathrm{d}t + \mathbf{B}(\phi_1, \phi_2, t)\,\mathrm{d}\vec{W}_t, \tag{SI.10}$$

where $\mathbf{A}(\phi_1, \phi_2, t)$ is given by

$$\mathbf{A}(\phi_1, \phi_2, t) = -\begin{pmatrix} b\left(1 - 2\frac{\phi_1}{k} + \frac{\phi_2}{k}\right) - d_1 & b\left(\frac{\phi_1}{k} - 1\right) + d_1 - d_2 \\ h(t) & -h(t) - d_2 \end{pmatrix}. \tag{SI.11}$$

We note that the argument of $\mathbf{B}$ in Eq. (SI.10) is now given by ϕ_1 and ϕ_2, so that the noise is additive rather than multiplicative.

We are interested in the variation of the total population size from the deterministic path $\left\langle {\xi_{1_t}}^2 \right\rangle$; we remark that by construction $\langle \xi_{1_t} \rangle = \langle \xi_{2_t} \rangle = 0$. The variances and covariance of ξ_{1_t} and ξ_{2_t} can be seen to evolve in time as follows[6]

$$\frac{\mathrm{d}\left\langle {\xi_{1_t}}^2 \right\rangle}{\mathrm{d}t} = 2A_{11}\left\langle {\xi_{1_t}}^2 \right\rangle + 2A_{12}\langle \xi_{1_t}\xi_{2_t} \rangle + (B_{11})^2 + (B_{12})^2, \tag{SI.12a}$$

$$\frac{\mathrm{d}\left\langle {\xi_{2_t}}^2 \right\rangle}{\mathrm{d}t} = 2A_{22}\left\langle {\xi_{2_t}}^2 \right\rangle + 2A_{21}\langle \xi_{1_t}\xi_{2_t} \rangle + (B_{22})^2 + (B_{21})^2, \tag{SI.12b}$$

$$\frac{\mathrm{d}\langle \xi_{1_t}\xi_{2_t} \rangle}{\mathrm{d}t} = A_{21}\left\langle {\xi_{1_t}}^2 \right\rangle + A_{12}\left\langle {\xi_{2_t}}^2 \right\rangle + (A_{11} + A_{22})\langle \xi_{1_t}\xi_{2_t} \rangle + B_{11}B_{21} + B_{12}B_{22}. \tag{SI.12c}$$

For a given set of parameters, we numerically integrate the five coupled Eqs. (SI.9) and Eqs. (SI.12). This provides the mean and covariance matrix for the bivariate Gaussian distribution of the number of normal and doomed cells as a function of time. For Approximation 1, the time t^* is defined by $\phi_1(t^*) = \ell$; this is the point in time when the total number of functional cells crosses the threshold for onset of NTC. The variance of the number of functional cells at this time is given by $\Sigma^2(t^*) = \left\langle {\xi_{1_{t^*}}}^2 \right\rangle$ within the LNA. We then use Eq. (20), where $\mu(\ell, t^*)$ is to be replaced by the right-hand side of Eq. (SI.9a), evaluated at t^*.

Approximation 2 is computed using Eq. (30), replacing $\phi(t)$ by $\phi_1(t)$, and $\Sigma^2(t)$ by $\left\langle {\xi_{1_t}}^2 \right\rangle$, respectively.

A comparison of Approximations 1 and 2

Fig. SI.1 shows a quantitative comparison of the distributions of first-passage time from the two approximations for Model 1. It indicates that Approximation 2 provides an improvement relative to Approximation 1. Both methods do considerably better than the deterministic approximation in Stocks et al.[5].

To compare the three approximations we have used the Earth Mover's Distance (EMD), also known as the Kantorovich metric[7], as a measure of distance between two probability distributions. Intuitively, it is a measure of the amount of 'effort' needed to turn one distribution into the other; it is the amount of probability that needs to be moved weighted by the distance it has to be moved. We choose this rather than, say, the Kullback–Leibler divergence[8] or total variation distance since the distribution of first-passage times from the deterministic approach is a Dirac delta-distribution[5] which results in infinite Kullback–Leibler divergence. The EMD gives a more useful measure of error.

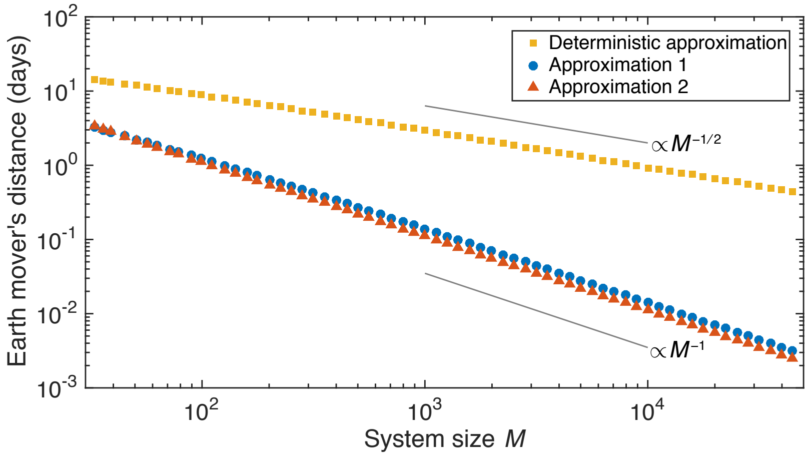

Figure SI.1. Measure of error for the predictions of NTCP for Model 1 using the Earth Mover's Distance (EMD)[7]. Each set of symbols shows the EMD of the distribution of first-passage times obtained from the different approximations relative to the distribution obtained by numerical integration of the master equation. We compare three approximations: the deterministic approximation from Stocks et al.[5] (i.e., the distribution of first-passage times is a delta-peak at the deterministic crossing time t^*), and Approximations 1 and 2 as described in the text. Results are shown as a function of the population-size parameter M. The data indicates that the EMD of Approximations 1 and 2 from the original model, Model 1, scales as M^{-1} with the typical size of the population; similar scaling is also observed using the Kullback–Leibler divergence (not shown). For the deterministic approximation the EMD decays much more slowly with the system-size parameter ($\propto M^{-1/2}$).

Complication-free tumour control

The objective of radiation therapy is to successfully eliminate cancerous cells while avoiding further complications from damaging normal tissue cells. We have developed analytical approximations for the efficient calculation of NTCPs. Tumour control probabilities—the probability of eliminating all cancer cells—from a stochastic birth-death model have been previously considered by Zaider and Minerbo[9]; the authors derive a general equation for the probability of the elimination of all tumour cells. Here, we combine these two results for NTCP and TCP to investigate how, in principle, mathematical models can be used to optimise the application of radiation therapy to achieve complication-free tumour control.

Model definitions

We consider a model, which we call Model 3, that contains both normal cells $\mathscr{N}$ and cancerous cells $\mathscr{C}$. The two populations are assumed to be spatially separated from each other. The normal cells are as described in Model 1. Cancerous cells, on the other hand, undergo mitosis with a constant rate b_2[9]; numerical evidence suggests that the resulting exponential growth characterise tumours of small sizes well[10]. Cancerous cells are also subject to a natural death with a rate d_2 and to death from a

source of radiation with hazard function $h_2(t)$. The model can be summarised by the following reactions:

$$\begin{aligned}
&\mathscr{N} \xrightarrow{b_1\left(1-\frac{N}{kM}\right)} \mathscr{N}+\mathscr{N}, \qquad && \mathscr{C} \xrightarrow{b_2} \mathscr{C}+\mathscr{C}, \qquad && \text{(mitosis)},\\
&\mathscr{N} \xrightarrow{d_1} \emptyset, && \mathscr{C} \xrightarrow{d_2} \emptyset, && \text{(natural death)},\\
&\mathscr{N} \xrightarrow{h_1(t)} \emptyset, && \mathscr{C} \xrightarrow{h_2(t)} \emptyset, && \text{(irradiated death)}.
\end{aligned} \tag{SI.13}$$

Although both types of cell are subject to the same source of radiation, the hazard functions $h_1(t)$ and $h_2(t)$ can differ. This is because each cell type differs in its susceptibility to radiation and in their ability to repair damaged DNA. We again consider the case of brachytherapy; the hazard function is as in Eq. (29), where the parameters $\alpha_{1,2}$, $\beta_{1,2}$, and $\gamma_{1,2}$ depend on the cell type. We also assume that, due to the presumed spatial separation of normal tissue and cancerous cells, the treatment can be targeted such that each cell type absorbs a different fraction of the total dose rate. This is incorporated into the hazard function by replacing the initial dose rate R_0 with an effective dose rate $\theta_{1,2}R_0$. The parameters describing the initial dose rate R_0 and the decay rate λ are characteristics of the radioactive implant and are thus common to the hazard function of both cell types. As before, we initialise the population of normal cells in its stationary state. We let there be initially C_0 cancer cells.

Tumour control probability, normal-tissue complication probability, and probability of complication-free control

The probability as a function of time of eliminating all cancer cells, $\mathrm{TCP}(t)$, is mathematically also a first-passage time problem. Zaider and Minerbo[9] developed an analytical description for TCP for the linear dynamics of cancerous cells described above. This was achieved using a generating-function. This approach is feasible due to two features of the problem: (i) the model is linear (i.e., cells do not interact with each other), and (ii) the boundary of interest for TCP is at zero (i.e., extinction of tumour cells). Their result for $\mathrm{TCP}(t)$ is[9]

$$\mathrm{TCP}(t) = \left[1 - \frac{C(t)/C_0}{1+b_2\int_0^t \mathrm{d}t' \frac{C(t)}{C(t')}}\right]^{C_0}, \tag{SI.14}$$

where $C(t)$ is the deterministic path for the expected number of cancerous cells, given by

$$\frac{\mathrm{d}C}{\mathrm{d}t} = [b_2 - d_2 - h_2(t)]\,C(t). \tag{SI.15}$$

While Eq. (SI.15) cannot be solved analytically in most cases, it can be integrated numerically for an efficient calculation of $\mathrm{TCP}(t)$.

Complication-free tumour control (CFC) refers to the elimination of all cancer cells while maintaining enough normally functioning tissue for an organ to operate without complications[11]. The probability of CFC as a function of time is therefore given by[11]

$$\mathrm{CFC}(t) = \mathrm{TCP}(t)\,[1-\mathrm{NTCP}(t)]. \tag{SI.16}$$

We remark that Eq. (SI.16) implies an equal weighting of the importance of tumour control and NTCs. In the most extreme cases, for example where NTCs relate to organ failure this is justified. In other cases, for example when NTC refers to increased urinal frequency, a complication may be preferable to a potentially life-threatening tumour. In such cases, Eq. (SI.16) can be modified by appropriately weighting the two probabilities to maximise a 'quality of life' measure in accordance with clinical experience[12].

Fig. SI.2 (a) shows the probabilities $1-\mathrm{NTCP}(t)$ and $\mathrm{TCP}(t)$ for Model 3 and for a specific choice of parameters (see Table SI.1). These quantities are obtained by Approximation 2 for NTCP, and Eq. (SI.14) for TCP. Similarly, Fig. SI.2 (b) shows $\mathrm{CFC}(t)$ and compares the results from our approximation to those of numerical integration of the master equation. For this choice of parameters we find a non-trivial time (~ 20 days) which maximises the probability of CFC. In the case of a temporary brachytherapy implant, this would indicate the optimum moment for removal.

In order to achieve CFC, we assume we are able to control the initial dose rate R_0 (i.e., the size of the radioactive seed) and the time at which the implant is removed. Fig. SI.2 (c) shows the probability of CFC for different values of time and initial dose, again efficiently generated using Approximation 2 for NTCP and Eq. (SI.14) for TCP. With the exception of the population sizes, the parameters we choose here were previously used to model the treatment of prostate carcinoma[5] consistent with experimentally collected parameters[13]. In this context NTC refers to acute radiation proctitis[14]. For these parameters,

the optimal strategy involves an initial dose of size 1.7 Gy and removal at a time over 50 days. Using this initial dose, the probability of CFC(t) does not decrease at large times, providing a large window for the removal of the implant or allowing the use of a permanent implant. This is not the case for all parameters; the optimum strategy may require the timely removal of the implant. An example of this is shown in Fig. SI.2 (d), which shows CFC(t) for parameters where the cancer cells have a three-fold higher growth rate than normal cells. The probability of CFC is peaked when implanting a high dose of radiation for a short time. For this case, we see the band where CFC is likely is narrow, indicating that such a treatment may be very sensitive to the time of removal of the implant.

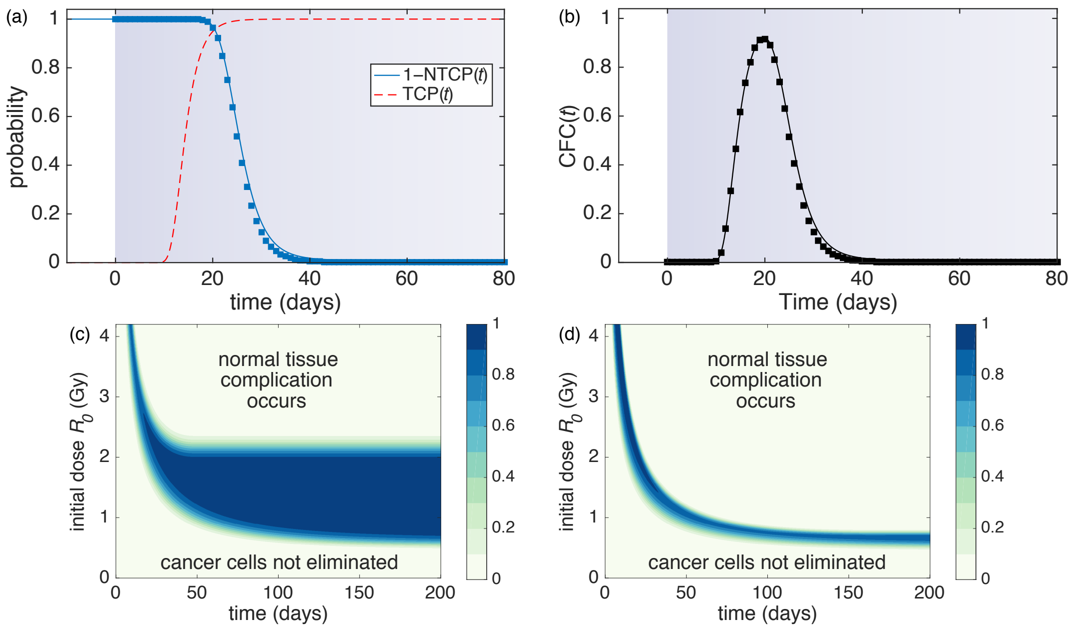

Figure SI.2. TCP, NTCP and probability of CFC for Model 3. **(a)** Probability that normal tissue complication has not yet occurred, $1-\mathrm{NTCP}(t)$, as predicted by Approximation 2 (blue line) and from numerical integration of the master equation (blue squares). Probability that the tumour is successfully eliminated TCP(t) (dashed red line). TCP is calculated as in Eq. (SI.14)[9]. The shading of the background indicates the hazard function $h(t)$. Initial dose $R_0 = 2.5$Gy. **(b)** Resulting probability of complication-free tumour control CFC(t). Black line is using Approximation 2 for NTCP and TCP as in Eq. (SI.14)[9]; results from direct numerical integration of the master equation are shown as black squares. **(c) and (d)** CFC(t) for different values of the initial dose R_0, and for two different sets of model parameters (see Table SI.1).

Case	b day^{-1}	d day^{-1}	α Gy^{-1}	β Gy^{-2}	γ day^{-1}	threshold for NTC ℓ	frac. of dose absorbed θ	pop. size
TCP Fig. SI.2 (a–c)	0.0165	0.0015	0.2	0.05	8.35		1.0	$C_0 = 10^3$
NTCP Fig. SI.2 (a–c)	0.055	0.005	0.1	0.01	8.35	0.5	0.2	$M = 10^3$
TCP Fig. SI.2 (d)	0.02	0.005	0.2	0.05	2.27		1.0	$C_0 = 10^3$
NTCP Fig. SI.2 (d)	0.0067	0.0017	0.1	0.01	2.27	0.2	0.4	$M = 10^3$

Table SI.1. Parameters used in Fig. SI.2, along with $\lambda = 0.0117$ day^{-1}. The parameters in the upper two rows were previously used to model brachytherapy as a treatment for prostate cancer, where the normal tissue complication refers to rectal proctitis[5]. The parameters in the bottom row are hypothetical, used to show that a change in the optimum treatment strategy may result upon variation of parameters.

References

1. Brenner, D. J. & Herbert, D. E. The use of the linear-quadratic model in clinical radiation oncology can be defended on the basis of empirical evidence and theoretical argument. *Med. physics* **24**, 1245–1248 (1997).
2. Brenner, D. J. The linear-quadratic model is an appropriate methodology for determining isoeffective doses at large doses per fraction. In *Seminars in radiation oncology*, vol. 18, 234–239 (Elsevier, 2008).

3. Fowler, J. F. 21 years of biologically effective dose. *The Br. journal radiology* **83**, 554–568 (2010).

4. Lea, D. & Catcheside, D. The mechanism of the induction by radiation of chromosome aberrations intradescantia. *J. genetics* **44**, 216–245 (1942).

5. Stocks, T., Hillen, T., Gong, J. & Burger, M. A stochastic model for the normal tissue complication probability (ntcp) and applications. *Math. medicine biology: a journal IMA* **34**, 469–492 (2016).

6. Risken, H. & Frank, T. *The Fokker-Planck Equation: Methods of Solution and Applications* (Springer-Verlag Berlin Heidelberg, 1996).

7. Gibbs, A. L. & Su, F. E. On choosing and bounding probability metrics. *Int. statistical review* **70**, 419–435 (2002).

8. Kullback, S. & Leibler, R. A. On information and sufficiency. *The annals mathematical statistics* **22**, 79–86 (1951).

9. Zaider, M. & Minerbo, G. Tumour control probability: a formulation applicable to any temporal protocol of dose delivery. *Phys. Medicine & Biol.* **45**, 279 (2000).

10. McAneney, H. & O'Rourke, S. Investigation of various growth mechanisms of solid tumour growth within the linear-quadratic model for radiotherapy. *Phys. Medicine & Biol.* **52**, 1039 (2007).

11. Mundt, A. J. & Roeske, J. C. *Intensity modulated radiation therapy: a clinical perspective* (PMPH-USA, 2005).

12. Kallman, P., Lind, B. K. & Brahme, A. An algorithm for maximizing the probability of complication-free tumour control in radiation therapy. *Phys. Medicine & Biol.* **37**, 871 (1992).

13. Carlson, D. J. *et al.* Comparison of in vitro and in vivo α/β ratios for prostate cancer. *Phys. Medicine & Biol.* **49**, 4477 (2004).

14. Kishan, A. U. & Kupelian, P. A. Late rectal toxicity after low-dose-rate brachytherapy: incidence, predictors, and management of side effects. *Brachytherapy* **14**, 148–159 (2015).
